# Characterising the Behavioural Determinants Associated with Developing Antipsychotic-Induced Metabolic Side Effects

**DOI:** 10.1192/bjo.2026.11251

**Published:** 2026-06-30

**Authors:** Emma Good, Sion Scott, Debi Bhattacharya, Sam Tromans, Michelle O’Reilly

**Affiliations:** 1 University of Leicester, Leicester, United Kingdom; 2 University of East Anglia, Norwich, United Kingdom

## Abstract

**Aims:**

Antipsychotics are the mainstay of treatment for severe mental illness (SMI) but cause metabolic side effects, such as rapid and clinically significant weight gain, hyperglycaemia, and hypercholesterolaemia, increasing the risk of type 2 diabetes and cardiovascular disease. Metabolic side effects develop through physiological mechanisms (e.g. plasma glucose dysregulation), but also through behaviour changes (e.g. increased calorie intake, sedentary lifestyle). This study aimed to characterise the behavioural determinants associated with developing metabolic side effects by exploring people’s experiences of taking antipsychotics for an SMI.

**Methods:**

Participants (n*=*22) were recruited, as members of the public via Mental Health charities and an existing database of people who had been previously prescribed antipsychotic treatment for an SMI (n*=*20), and via National Health Service Trusts who were recently initiated on antipsychotic treatment for an SMI (n*=*2). Sociodemographic characteristics (age group, biological sex, ethnic group, index of multiple deprivation) were collected from participants to ensure a diverse sample. Individual, reflective interviews with a semi-structured style were undertaken to explore determinants associated with changes in behaviour when antipsychotics were initiated. Template analysis was undertaken, comprising an inductive approach and a priorithemes.

**Results:**

The following main themes were created which were associated with changes in behaviour influencing the development of metabolic side effects: environmental determinants (e.g. Covid-19 pandemic, weather, finances); social determinants (e.g. social exclusion, social support, provision of food from family/friends); emotional determinants (e.g. experience of trauma, low mood, shame); and motivation around healthy eating and physical activity (e.g.convenience, cravings, self-medication). Integrative themes included: impact of weight gain (e.g. on identity, quality of life, comorbidities); experience of being a mental health patient (e.g. clinical support, monitoring of side effects); and impact of medication/side effects on eating and physical activity behaviours (e.g. sedation, decreased energy, prior knowledge of side effects).

**Conclusion:**

There are a variety of determinants influencing behaviours associated with developing antipsychotic-induced metabolic side effects. Current clinical practice is to treat side effects with pharmacological and/or non-pharmacological interventions once they have developed as per National Institute for Health and Care Excellence Guidelines and the Lester Tool. Interventions targeting specific behaviours and determinants should be implemented at the start of antipsychotic treatment to prevent metabolic side effects from developing. Further research should explore interventions and ongoing support appropriate to, and developed in partnership with, this population.

This study was sponsored by a University of Leicester PhD scholarship and Pharmacy Research UK.

